# Crystal structure of (7-{[bis­(pyridin-2-ylmeth­yl)amino-κ^3^ *N*,*N*′,*N*′′]meth­yl}-5-chloro­quinolin-8-ol)di­bromidozinc(II)

**DOI:** 10.1107/S2056989022001530

**Published:** 2022-02-15

**Authors:** Koji Kubono, Yukiyasu Kashiwagi, Keita Tani, Kunihiko Yokoi

**Affiliations:** a Osaka Kyoiku University, 4-698-1 Asahigaoka, Kashiwara, Osaka 582-8582, Japan; b Osaka Research Institute of Industrial Science and Technology, 1-6-50 Morinomiya, Joto-ku, Osaka 536-8553, Japan

**Keywords:** crystal structure, zinc(II) complex, 8-quinolinol, bis­(2-picoly)amine, C—H⋯Br inter­actions

## Abstract

In the title compound, [ZnBr_2_(C_22_H_19_ClN_4_O)], the Zn^II^ atom adopts a distorted square-pyramidal coordination geometry, formed by two bromido ligands and three N atoms of the bis­(pyridin-2-ylmeth­yl)amine moiety in the penta­dentate ligand containing quinolinol. The Zn^II^ atom is located well above the mean basal plane of the square-based pyramid. The apical position is occupied by a Br atom. The O and N atoms of the quinolinol moiety in the ligand are not coordinated to the Zn^II^ atom. An intra­molecular O—H⋯N hydrogen bond, generating an *S*(5) ring motif, stabilizes the mol­ecular structure. In the crystal, the mol­ecules are linked by inter­molecular C—H⋯Br hydrogen bonds, generating ribbon structures containing alternating *R*_2_^2^(22) and *R*_2_^2^(14) rings. These ribbons are linked through an inter­molecular C—H⋯Br hydrogen bond, forming a two-dimensional network sheet.

## Abstract 2

In the title compound, the Zn^II^ atom has a distorted square-pyramidal coordination environment. The mol­ecular structure exhibits an intra­molecular O—H⋯N hydrogen bond. In the crystal, the mol­ecules are linked by inter­molecular C—H⋯Br hydrogen bonds, generating ribbon structures. These ribbons are linked though inter­molecular C—H⋯Br hydrogen bonds, forming a two-dimensional network sheet.

## Chemical context

8-Quinolinol (Hq) is a notable bidentate ligand and an excellent analytical reagent for the determination of the concentration and separation of metal ions (Medlin, 1960 ▸; Eguchi *et al.*, 2019 ▸). Hq derivatives and their metal complexes have wide applications in diverse areas such as pharmaceuticals (Lai *et al.*, 2009 ▸) and organic light-emitting diodes (Li *et al.*, 2020 ▸). Bis(pyridin-2-ylmeth­yl)amine [di(2-picol­yl)amine, dpa] is a well-known tridentate ligand and highly selective for Zn^II^. Its derivatives are utilized as chemosensors for detecting Zn^II^ at low concentration in biological samples (Lin *et al.*, 2013 ▸) . In addition, some Zn^II^ complexes with dpa derivatives comprise a binding site for polyphosphates such as diphos­phate and adenosine triphosphate, and can act as respective anion sensors (Aoki *et al.*, 2020 ▸; Bazany-Rodríguez *et al.*, 2020 ▸). We, hence, developed the penta­dentate ligand, 7-{[bis(pyridin-2-ylmeth­yl)amino]­meth­yl}-5-chloro­quinolin-8-ol (HClqdpa) containing Hq and dpa moieties (Kubono *et al.*, 2015 ▸). Subsequently, reactions between HClqdpa and Zn^II^ salts were carried out in order to develop fluorescent anion sensors. In the course of these studies, a crystalline complex was obtained from the reaction with zinc(II) bromide. Here, the crystal structure of the respective title compound is reported.

## Structural commentary

**Figure 1:**
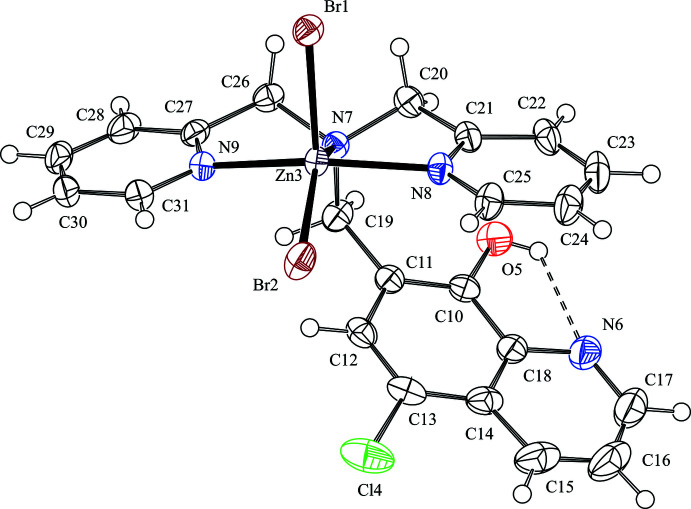
The mol­ecular structure of the title compound, with atom labeling. Displacement ellipsoids are drawn at the 50% probability level. H atoms are represented by spheres of arbitrary radius. The intra­molecular O—H⋯N hydrogen bond is shown as a dashed line.

The mol­ecular structure of the title compound is shown in Fig. 1 ▸. The Zn^II^ atom adopts a distorted square-pyramidal geometry and coordinates two bromido ligands (Br1 and Br2) and three N atoms (N7, N8 and N9) of the dpa moiety in HClqdpa forming the ZnBr_2_(dpa) unit. The Hq moiety of the penta­dentate ligand (HClqdpa) is not coordinated to the Zn^II^ center. The five-coordinate geometry parameter, *τ* = (*β* − *α*)/60, derived from the two largest angles (*α* < *β*) in a structure has ideal values of 0 for square-pyramidal and of 1 for trigonal–bipyramidal geometry (Addison *et al.*, 1984 ▸). In the title compound it is equal to 0.138. The Zn^II^ atom is located 0.5574 (3) Å above the mean basal plane (Br2/N8/N7/N9) of the square-based pyramid. The dpa moiety is meridionally bound to the Zn^II^ atom. The apical position is occupied by the Br1 atom with the apical bond being slightly elongated to 2.4419 (4) Å compared to the equatorial Br2—Zn3 bond length of 2.4085 (4) Å. The Zn—N bond lengths in the title compound are 2.1455 (18) and 2.1497 (18) Å for the pyridyl atoms (N8, N9), and 2.2670 (18) Å for the tertiary atom N7. In comparison, the Zn—N bond lengths in the crystal structure of a related complex with a mesityl methyl­ene-appended dpa derivative are 2.093 (3), 2.066 (3), and 2.521 (3) Å (MUDWEQ; Acharya *et al.*, 2020 ▸). The bond lengths for the pyridyl N atoms are, hence, shorter and the bond length for the tertiary N atom is longer than those in the title compound. The dihedral angle between the two pyridine rings in the title compound is 15.84 (13)°. In a related complex (MUDWEQ; Acharya *et al.*, 2020 ▸), this dihedral angle between two pyridine rings is widened to 23.53 (18)°, concomitant with an increased *τ* parameter of 0.211. The phenolic oxygen O5 of the Hq moiety is bound to hydrogen atom H5, which was found and refined freely. The proton, therefore, does not dissociate and no phen­oxy function is formed. There is an intra­molecular hydrogen bond, O5—H5⋯N6, generating an *S*(5) ring motif (Fig. 1 ▸ and Table 1 ▸). The quinoline ring system is slightly bent with an r.m.s. deviation of 0.018 (3) Å. In the quinoline ring system, the largest deviation from the mean plane is 0.020 (4) Å for carbon atom C15. The quinoline plane subtends dihedral angles of 24.14 (11) and 36.65 (11)° with the two pyridine rings. 
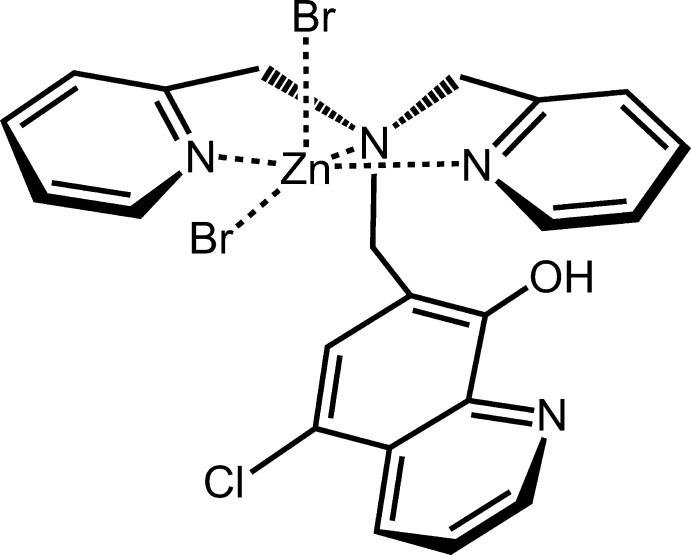


## Supra­molecular features

**Figure 2:**
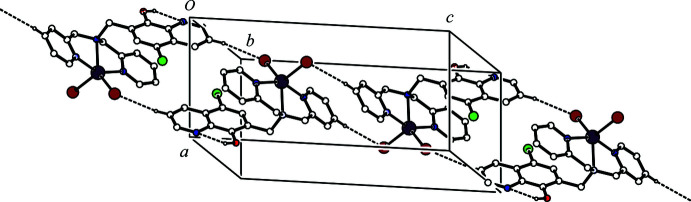
A portion of the crystal packing of the title compound showing the ribbon structure motif built from alternating 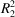 (22) and 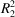 (14) rings. The C—H⋯Br hydrogen bonds between the dimers and the intra­molecular hydrogen bonds are shown as dashed lines. H atoms not involved in the inter­actions were omitted for clarity.

**Figure 3:**
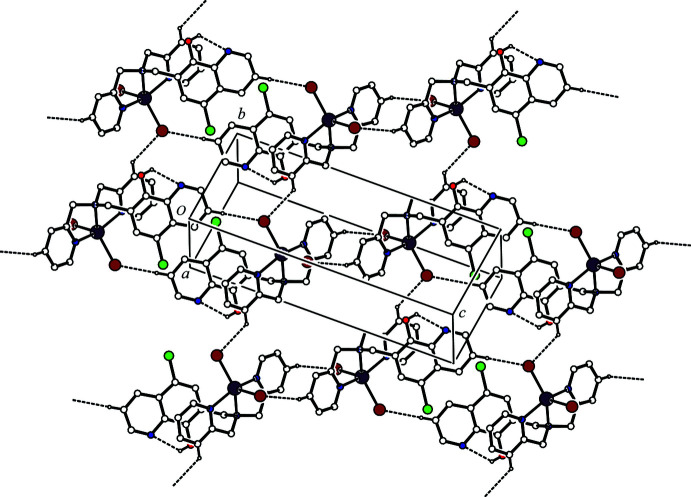
A packing diagram of the title compound showing the two-dimensional network sheet structure. The inter­molecular C—H⋯Br and intra­molecular O—H⋯N hydrogen bonds are shown as dashed lines. H atoms not involved in the inter­actions were omitted for clarity.

In the crystal, two mol­ecules are associated through a pair of inter­molecular C—H⋯Br hydrogen bonds [C16—H16⋯Br2^i^; symmetry code: (i) 1 − *x*, −*y*, −*z*] (Table 1 ▸), forming a centrosymmetric dimer with an 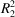 (22) ring motif. Another pair of inter­molecular C—H⋯Br hydrogen bonds is observed [C29—H29⋯Br1^iii^; symmetry code: (iii) 1 − *x*, 1 − *y*, 1 − *z*] (Table 1 ▸), which forms another centrosymmetric dimer with an 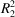 (14) ring motif. The different hydrogen-bonded pairs of mol­ecules are also linked to each other by these inter­molecular C—H⋯Br hydrogen bonds, generating a ribbon structure along [0 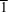 1] based on alternating 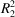 (22) and 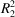 (14) hydrogen-bonding motifs (Fig. 2 ▸). In the crystal, mol­ecules are further linked by an inter­molecular C—H⋯Br hydrogen bond [C22—H22⋯Br2^ii^; symmetry code: (ii) *x* + 1, *y* − 1, *z*] (Table 1 ▸), forming a *C*(6) chain motif running along [2 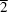 0] (Fig. 3 ▸). The ribbon structures are, therefore, linked through the inter­molecular C22—H22⋯Br2^ii^ hydrogen bonds and form a two-dimensional network sheet parallel to [22 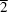 ] (Fig. 3 ▸).

## Database survey

A search of the Cambridge Structural Database (CSD, Version 5.42; May 2021; Groom *et al.*, 2016 ▸) using *ConQuest* (Bruno *et al.*, 2002 ▸) for Zn^II^ complexes with the [bis­(pyridin-2-ylmeth­yl)amino]­methyl fragment as ligand gave 517 hits, and among those, eight hits with two bromido ligands. Of these eight analogues, three structures are complexes with dpa bearing a tertiary N donor atom directly bound to an aromatic moiety (IRISEJ; Zhang *et al.*, 2016 ▸; ZEGZOC; Gao *et al.*, 2012 ▸; TORLUH; Plenio *et al.*, 1996 ▸). In the remaining five di­bromido Zn^II^ complexes with dpa derivatives (comprising four compounds), the tertiary N atoms are bound to aliphatic carbon atoms as in the title complex. Four of these five closely related structures exhibit square-pyramidal geometries with dpa being meridionally coordinated (YOZZOC; Abufarag *et al.*, 1995 ▸; RUVCUI; Škalamera *et al.*, 2016 ▸; MUDWEQ; Acharya *et al.*, 2020 ▸; IHIJIV; Juraj *et al.*, 2020 ▸). The remaining exceptional structure is *fac*-{*N*,*N′*-bis­[(pyridine-2-yl)meth­yl]propan-2-amine}­dibromido­zinc(II) (IHIJOB; Juraj *et al.*, 2020 ▸), which adopts a trigonal–bipyramidal geometry with dpa being facially coordinated. This structure is a polymorph of one complex with a more typical geometry mentioned above (IHIJIV; Juraj *et al.*, 2020 ▸). A search for mol­ecular structures containing Zn^II^ and the Hq moiety in which the H atom of the phenolic hy­droxy group is not dissociated gave 29 hits (comprising 25 compounds). Of these, six structures (three compounds) are ion-pairs between tetra­chlorido­zincate(II) and an 8-hy­droxy­quinolin-1-ium (H_2_q^+^) derivative, for example, (H_2_q)_2_[ZnCl_4_] (FARFIP; Lamshöft *et al.*, 2011 ▸). Eight structures are ion-pairs between H_2_q^+^ derivatives and anionic complexes consisting of Zn*X*_2_ (*X* = Cl, Br, or I) and quinolin-8-lato derivatives, *e.g.* 8-hy­droxy-2-methyl­quinolino­linium di­iodo­(2-methyl­uinolin-8-lato)zinc(II) (AYOCOH; Najafi *et al.*, 2011 ▸). Two structures are ion-pairs between H_2_q^+^ derivatives and anionic Zn^II^ complexes with other chelate ligands, *e.g.* bis­(8-hy­droxy­quinolin-1-ium) tris­(4-nitro­phenol) bis­(pyridine-2,6-carboxyl­ato)zinc(II) dihydrate (MIYKEN; Singh *et al.*, 2019 ▸). The remaining 13 structures (12 compounds) are Zn^II^ chelate complexes containing the Hq ligand with an undissociated phenolic functional group, *e.g.*, bis­(8-hy­droxy­quinolin-2-carboxyl­ato)zinc(II) trihydrate (QOCRAC; McDonald *et al.*, 2008 ▸). A crystal structure of a Zn^II^ complex containing the Hq moiety which is neither the counter-cation of an ion-pair nor bound to Zn^II^ has not been reported yet. A search for Zn^II^ complexes in which the entire ligand scaffold and substitution is also more analogous to the title compound, *i.e*. with [bis(pyridin-2-ylmeth­yl)amino]­methyl at the 2-position of Hq or respective derivatives, gave three hits (CIGJAF; Royzen *et al.*, 2013 ▸; RIZROI; Xue *et al.*, 2008 ▸; TEHDOA; Royzen *et al.*, 2006 ▸). In the three structures, the phenolic hy­droxy group is deprotonated and coordinated by Zn^II^.

## Synthesis and crystallization

The HClqdpa ligand (97.7 mg, 0.250 mmol) was dissolved in 15 mL of hot aceto­nitrile. Then a solution of zinc(II) bromide (56.4 mg, 0.250 mmol) in 15 mL of hot aceto­nitrile was added to the ligand solution. The mixture was stirred for 20 min at 333 K. After removal of the solvent at room temperature in air for one week, colorless crystals of the title compound were obtained (yield 35%; m.p. 496–497 K). Analysis calculated for C_22_H_19_Br_2_ClN_4_OZn: C 42.89, H 3.11, N 9.09%; found: C 42.94, H 3.02, N 8.95%.

## Refinement

Crystal data, data collection and structure refinement details are summarized in Table 2 ▸. The hy­droxy H atom was located in a difference-Fourier map and freely refined. The C-bound H atoms were positioned geometrically and refined using a riding model: C—H = 0.95–0.99 Å with *U*_iso_(H) = 1.2*U*_eq_(C). One outlier reflex (002) was omitted from the refinement.

## Supplementary Material

Crystal structure: contains datablock(s) global, I. DOI: 10.1107/S2056989022001530/yz2016sup1.cif

Structure factors: contains datablock(s) I. DOI: 10.1107/S2056989022001530/yz2016Isup2.hkl

CCDC reference: 2150991

Additional supporting information:  crystallographic information; 3D view; checkCIF report

## Figures and Tables

**Table 1:** Hydrogen-bond geometry (Å, °)

*D*—H⋯*A*	*D*—H	H⋯*A*	*D*⋯*A*	*D*—H⋯*A*
O5—H5⋯N6	0.79 (4)	2.14 (4)	2.653 (3)	124 (3)
C16—H16⋯Br2^i^	0.95	2.87	3.808 (3)	170
C22—H22⋯Br2^ii^	0.95	2.88	3.581 (3)	131
C29—H29⋯Br1^iii^	0.95	2.90	3.798 (3)	158

Symmetry codes: (i) 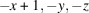 ; (ii) 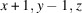 ; (iii) 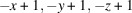 .

**Table 2:** Experimental details

Crystal data	
Chemical formula	[ZnBr_2_(C_22_H_19_ClN_4_O)]
*M*_r_	616.05
Crystal system, space group	Triclinic, *P* 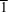
Temperature (K)	173
*a*, *b*, *c* (Å)	7.6779 (3), 8.7860 (4), 18.1379 (8)
α, β, γ (°)	89.460 (6), 89.617 (6), 66.878 (5)
*V* (Å^3^)	1125.21 (9)
*Z*	2
Radiation type	Mo *K*α
μ (mm^−1^)	4.78
Crystal size (mm)	0.35 × 0.20 × 0.15

Data collection	
Diffractometer	Rigaku R-AXIS RAPID
Absorption correction	Multi-scan (*ABSCOR*; Higashi, 1995 ▸)
*T*_min_, *T*_max_	0.316, 0.487
No. of measured, independent and observed [*F* ^2^ > 2.0σ(*F* ^2^)] reflections	11009, 5114, 4386
*R*_int_	0.017
(sin θ/λ)_max_ (Å^−1^)	0.648

Refinement	
*R*[*F* ^2^ > 2σ(*F* ^2^)], *wR*(*F* ^2^), *S*	0.027, 0.059, 1.07
No. of reflections	5114
No. of parameters	284
H-atom treatment	H atoms treated by a mixture of independent and constrained refinement
Δρ_max_, Δρ_min_ (e Å^−3^)	0.59, −0.65

Computer programs: *RAPID-AUTO* (Rigaku, 2006 ▸), *SIR92* (Altomare, *et al.*, 1993 ▸), *SHELXL2014/7* (Sheldrick, 2015 ▸), *PLATON* (Spek, 2020 ▸), and *CrystalStructure* (Rigaku, 2016 ▸).

## supplementary crystallographic information

### Crystal data

**Table:**

[ZnBr_2_(C_22_H_19_ClN_4_O)]	*Z* = 2
*M**_r_* = 616.05	*F*(000) = 608.00
Triclinic, *P*1	*D*_x_ = 1.818 Mg m^−^^3^
*a* = 7.6779 (3) Å	Mo *K*α radiation, λ = 0.71075 Å
*b* = 8.7860 (4) Å	Cell parameters from 9577 reflections
*c* = 18.1379 (8) Å	θ = 2.5–27.4°
α = 89.460 (6)°	µ = 4.78 mm^−^^1^
β = 89.617 (6)°	*T* = 173 K
γ = 66.878 (5)°	Block, colorless
*V* = 1125.21 (9) Å^3^	0.35 × 0.20 × 0.15 mm

### Data collection

**Table:**

Rigaku R-AXIS RAPID diffractometer	4386 reflections with *F*^2^ > 2.0σ(*F*^2^)
Detector resolution: 10.000 pixels mm^-1^	*R*_int_ = 0.017
ω scans	θ_max_ = 27.4°, θ_min_ = 2.8°
Absorption correction: multi-scan (ABSCOR; Higashi, 1995)	*h* = −9→9
*T*_min_ = 0.316, *T*_max_ = 0.487	*k* = −11→11
11009 measured reflections	*l* = −23→23
5114 independent reflections	

### Refinement

**Table:**

Refinement on *F*^2^	Secondary atom site location: difference Fourier map
*R*[*F*^2^ > 2σ(*F*^2^)] = 0.027	Hydrogen site location: inferred from neighbouring sites
*wR*(*F*^2^) = 0.059	H atoms treated by a mixture of independent and constrained refinement
*S* = 1.07	*w* = 1/[σ^2^(*F*_o_^2^) + (0.024*P*)^2^ + 0.8532*P*] where *P* = (*F*_o_^2^ + 2*F*_c_^2^)/3
5114 reflections	(Δ/σ)_max_ < 0.001
284 parameters	Δρ_max_ = 0.59 e Å^−^^3^
0 restraints	Δρ_min_ = −0.65 e Å^−^^3^
Primary atom site location: structure-invariant direct methods	

### Special details

**Table:**

Geometry. All esds (except the esd in the dihedral angle between two l.s. planes) are estimated using the full covariance matrix. The cell esds are taken into account individually in the estimation of esds in distances, angles and torsion angles; correlations between esds in cell parameters are only used when they are defined by crystal symmetry. An approximate (isotropic) treatment of cell esds is used for estimating esds involving l.s. planes.
Refinement. Refinement was performed using all reflections. The weighted R-factor (wR) and goodness of fit (S) are based on F^2^. R-factor (gt) are based on F. The threshold expression of F^2^ > 2.0 sigma(F^2^) is used only for calculating R-factor (gt).

### Fractional atomic coordinates and isotropic or equivalent isotropic displacement parameters (Å^2^)

**Table:**

	*x*	*y*	*z*	*U*_iso_*/*U*_eq_	
Br1	0.27928 (3)	0.13134 (3)	0.42388 (2)	0.02925 (7)	
Br2	0.21673 (4)	0.34246 (3)	0.22122 (2)	0.03710 (7)	
Zn3	0.43550 (3)	0.18902 (3)	0.31584 (2)	0.02169 (7)	
Cl4	0.52345 (11)	0.31327 (11)	0.04318 (5)	0.0578 (2)	
O5	1.0962 (2)	−0.2077 (2)	0.21912 (11)	0.0350 (4)	
N6	1.0498 (3)	−0.2729 (3)	0.07984 (13)	0.0399 (5)	
N7	0.7489 (3)	0.0783 (2)	0.34256 (10)	0.0226 (4)	
N8	0.5357 (3)	−0.0510 (2)	0.26615 (11)	0.0249 (4)	
N9	0.4920 (3)	0.3899 (2)	0.36144 (10)	0.0252 (4)	
C10	0.9635 (3)	−0.0864 (3)	0.17935 (13)	0.0259 (5)	
C11	0.8577 (3)	0.0643 (3)	0.21048 (13)	0.0248 (5)	
C12	0.7210 (3)	0.1857 (3)	0.16621 (14)	0.0294 (5)	
H12	0.649031	0.290977	0.186741	0.035*	
C13	0.6895 (4)	0.1560 (3)	0.09501 (15)	0.0337 (5)	
C14	0.7943 (4)	0.0008 (3)	0.06192 (14)	0.0337 (6)	
C15	0.7731 (5)	−0.0458 (4)	−0.01112 (16)	0.0489 (8)	
H15	0.679789	0.029174	−0.042671	0.059*	
C16	0.8870 (5)	−0.1980 (5)	−0.03553 (17)	0.0595 (10)	
H16	0.873842	−0.230341	−0.084318	0.071*	
C17	1.0237 (5)	−0.3070 (4)	0.01133 (17)	0.0521 (8)	
H17	1.102771	−0.412396	−0.007394	0.063*	
C18	0.9343 (3)	−0.1194 (3)	0.10505 (13)	0.0297 (5)	
C19	0.8847 (3)	0.1035 (3)	0.28896 (13)	0.0291 (5)	
H19A	1.015382	0.032903	0.304228	0.035*	
H19B	0.870946	0.220136	0.291434	0.035*	
C20	0.7877 (3)	−0.0985 (3)	0.35371 (14)	0.0278 (5)	
H20A	0.736052	−0.114214	0.402007	0.033*	
H20B	0.926284	−0.163249	0.354144	0.033*	
C21	0.6994 (3)	−0.1604 (3)	0.29331 (13)	0.0255 (5)	
C22	0.7819 (4)	−0.3211 (3)	0.26675 (15)	0.0334 (6)	
H22	0.896330	−0.397844	0.287479	0.040*	
C23	0.6955 (4)	−0.3673 (3)	0.21001 (17)	0.0401 (6)	
H23	0.751460	−0.475780	0.190356	0.048*	
C24	0.5265 (4)	−0.2547 (3)	0.18174 (16)	0.0396 (6)	
H24	0.464442	−0.284338	0.142650	0.048*	
C25	0.4499 (3)	−0.0976 (3)	0.21191 (14)	0.0325 (5)	
H25	0.332682	−0.020331	0.193508	0.039*	
C26	0.7580 (3)	0.1610 (3)	0.41217 (12)	0.0260 (5)	
H26A	0.891471	0.138494	0.424156	0.031*	
H26B	0.703999	0.117546	0.452978	0.031*	
C27	0.6470 (3)	0.3453 (3)	0.40353 (12)	0.0258 (5)	
C28	0.6961 (4)	0.4618 (3)	0.43949 (14)	0.0349 (6)	
H28	0.808069	0.428428	0.468254	0.042*	
C29	0.5782 (5)	0.6278 (3)	0.43250 (15)	0.0412 (7)	
H29	0.607517	0.709633	0.457163	0.049*	
C30	0.4187 (4)	0.6729 (3)	0.38959 (15)	0.0380 (6)	
H30	0.336091	0.785945	0.384380	0.046*	
C31	0.3804 (4)	0.5510 (3)	0.35418 (14)	0.0327 (5)	
H31	0.271613	0.582321	0.323689	0.039*	
H5	1.131 (6)	−0.285 (5)	0.193 (2)	0.071 (13)*	

### Atomic displacement parameters (Å^2^)

**Table:**

	*U*^11^	*U*^22^	*U*^33^	*U*^12^	*U*^13^	*U*^23^
Br1	0.02994 (12)	0.03405 (13)	0.02819 (12)	−0.01729 (10)	0.00204 (9)	−0.00401 (9)
Br2	0.03370 (13)	0.03205 (13)	0.03195 (13)	0.00201 (10)	−0.00934 (10)	−0.00635 (10)
Zn3	0.01991 (12)	0.01955 (12)	0.02461 (13)	−0.00657 (10)	−0.00128 (10)	−0.00394 (10)
Cl4	0.0467 (4)	0.0647 (5)	0.0546 (5)	−0.0144 (4)	−0.0121 (4)	0.0322 (4)
O5	0.0269 (9)	0.0347 (10)	0.0364 (10)	−0.0046 (8)	0.0016 (8)	0.0014 (8)
N6	0.0514 (14)	0.0411 (13)	0.0359 (12)	−0.0276 (11)	0.0146 (11)	−0.0090 (10)
N7	0.0225 (9)	0.0236 (9)	0.0229 (9)	−0.0104 (8)	−0.0006 (7)	−0.0011 (7)
N8	0.0220 (9)	0.0220 (9)	0.0308 (10)	−0.0089 (8)	0.0037 (8)	−0.0054 (8)
N9	0.0297 (10)	0.0228 (9)	0.0243 (10)	−0.0114 (8)	0.0001 (8)	−0.0022 (8)
C10	0.0246 (11)	0.0282 (11)	0.0273 (12)	−0.0131 (10)	0.0011 (9)	0.0033 (9)
C11	0.0240 (11)	0.0275 (11)	0.0266 (11)	−0.0140 (9)	0.0021 (9)	−0.0002 (9)
C12	0.0289 (12)	0.0257 (12)	0.0354 (13)	−0.0128 (10)	0.0030 (10)	0.0036 (10)
C13	0.0309 (12)	0.0373 (14)	0.0351 (13)	−0.0161 (11)	−0.0043 (11)	0.0146 (11)
C14	0.0398 (14)	0.0455 (15)	0.0274 (12)	−0.0295 (13)	−0.0022 (11)	0.0052 (11)
C15	0.0577 (19)	0.074 (2)	0.0294 (14)	−0.0417 (18)	−0.0053 (13)	0.0042 (14)
C16	0.082 (2)	0.087 (3)	0.0349 (16)	−0.060 (2)	0.0105 (16)	−0.0206 (17)
C17	0.072 (2)	0.0547 (19)	0.0442 (17)	−0.0404 (18)	0.0201 (16)	−0.0204 (15)
C18	0.0347 (13)	0.0332 (13)	0.0286 (12)	−0.0216 (11)	0.0066 (10)	−0.0022 (10)
C19	0.0277 (12)	0.0354 (13)	0.0284 (12)	−0.0167 (10)	0.0039 (9)	−0.0059 (10)
C20	0.0231 (11)	0.0241 (11)	0.0335 (13)	−0.0063 (9)	−0.0015 (10)	0.0020 (10)
C21	0.0226 (10)	0.0211 (11)	0.0334 (12)	−0.0092 (9)	0.0071 (9)	−0.0021 (9)
C22	0.0290 (12)	0.0198 (11)	0.0488 (16)	−0.0067 (10)	0.0130 (11)	−0.0038 (11)
C23	0.0406 (15)	0.0268 (13)	0.0563 (17)	−0.0169 (12)	0.0190 (13)	−0.0170 (12)
C24	0.0438 (15)	0.0392 (15)	0.0447 (16)	−0.0255 (13)	0.0087 (12)	−0.0165 (12)
C25	0.0287 (12)	0.0323 (13)	0.0389 (14)	−0.0142 (11)	0.0032 (11)	−0.0102 (11)
C26	0.0251 (11)	0.0328 (12)	0.0225 (11)	−0.0137 (10)	−0.0033 (9)	−0.0009 (9)
C27	0.0313 (12)	0.0322 (12)	0.0200 (10)	−0.0189 (10)	0.0046 (9)	−0.0044 (9)
C28	0.0456 (15)	0.0472 (15)	0.0254 (12)	−0.0327 (13)	0.0033 (11)	−0.0065 (11)
C29	0.0684 (19)	0.0396 (15)	0.0326 (14)	−0.0393 (15)	0.0181 (13)	−0.0140 (11)
C30	0.0573 (17)	0.0252 (12)	0.0356 (14)	−0.0206 (12)	0.0143 (13)	−0.0059 (10)
C31	0.0415 (14)	0.0252 (12)	0.0313 (13)	−0.0129 (11)	0.0049 (11)	−0.0017 (10)

### Geometric parameters (Å, º)

**Table:**

Br1—Zn3	2.4419 (4)	C16—C17	1.396 (5)
Br2—Zn3	2.4085 (4)	C16—H16	0.9500
Zn3—N8	2.1455 (18)	C17—H17	0.9500
Zn3—N9	2.1497 (18)	C19—H19A	0.9900
Zn3—N7	2.2670 (18)	C19—H19B	0.9900
Cl4—C13	1.740 (3)	C20—C21	1.506 (3)
O5—C10	1.355 (3)	C20—H20A	0.9900
O5—H5	0.79 (4)	C20—H20B	0.9900
N6—C17	1.316 (4)	C21—C22	1.390 (3)
N6—C18	1.371 (3)	C22—C23	1.376 (4)
N7—C20	1.474 (3)	C22—H22	0.9500
N7—C26	1.478 (3)	C23—C24	1.385 (4)
N7—C19	1.498 (3)	C23—H23	0.9500
N8—C21	1.341 (3)	C24—C25	1.387 (3)
N8—C25	1.341 (3)	C24—H24	0.9500
N9—C27	1.339 (3)	C25—H25	0.9500
N9—C31	1.342 (3)	C26—C27	1.512 (3)
C10—C11	1.377 (3)	C26—H26A	0.9900
C10—C18	1.419 (3)	C26—H26B	0.9900
C11—C12	1.414 (3)	C27—C28	1.391 (3)
C11—C19	1.502 (3)	C28—C29	1.386 (4)
C12—C13	1.362 (4)	C28—H28	0.9500
C12—H12	0.9500	C29—C30	1.374 (4)
C13—C14	1.420 (4)	C29—H29	0.9500
C14—C18	1.409 (4)	C30—C31	1.381 (3)
C14—C15	1.419 (4)	C30—H30	0.9500
C15—C16	1.355 (5)	C31—H31	0.9500
C15—H15	0.9500		
			
N8—Zn3—N9	149.88 (7)	C14—C18—C10	120.6 (2)
N8—Zn3—N7	76.13 (7)	N7—C19—C11	114.29 (18)
N9—Zn3—N7	75.20 (7)	N7—C19—H19A	108.7
N8—Zn3—Br2	98.53 (5)	C11—C19—H19A	108.7
N9—Zn3—Br2	98.16 (5)	N7—C19—H19B	108.7
N7—Zn3—Br2	141.63 (5)	C11—C19—H19B	108.7
N8—Zn3—Br1	98.76 (5)	H19A—C19—H19B	107.6
N9—Zn3—Br1	97.48 (5)	N7—C20—C21	110.67 (19)
N7—Zn3—Br1	105.26 (5)	N7—C20—H20A	109.5
Br2—Zn3—Br1	113.102 (14)	C21—C20—H20A	109.5
C10—O5—H5	104 (3)	N7—C20—H20B	109.5
C17—N6—C18	116.6 (3)	C21—C20—H20B	109.5
C20—N7—C26	112.22 (18)	H20A—C20—H20B	108.1
C20—N7—C19	111.92 (18)	N8—C21—C22	121.6 (2)
C26—N7—C19	108.08 (16)	N8—C21—C20	116.01 (19)
C20—N7—Zn3	102.79 (13)	C22—C21—C20	122.4 (2)
C26—N7—Zn3	102.85 (13)	C23—C22—C21	119.1 (2)
C19—N7—Zn3	118.69 (14)	C23—C22—H22	120.5
C21—N8—C25	119.2 (2)	C21—C22—H22	120.5
C21—N8—Zn3	114.94 (14)	C22—C23—C24	119.5 (2)
C25—N8—Zn3	125.90 (16)	C22—C23—H23	120.2
C27—N9—C31	119.2 (2)	C24—C23—H23	120.2
C27—N9—Zn3	115.33 (15)	C23—C24—C25	118.3 (2)
C31—N9—Zn3	125.38 (16)	C23—C24—H24	120.8
O5—C10—C11	120.9 (2)	C25—C24—H24	120.8
O5—C10—C18	118.3 (2)	N8—C25—C24	122.3 (2)
C11—C10—C18	120.8 (2)	N8—C25—H25	118.9
C10—C11—C12	118.2 (2)	C24—C25—H25	118.9
C10—C11—C19	122.2 (2)	N7—C26—C27	109.09 (18)
C12—C11—C19	119.6 (2)	N7—C26—H26A	109.9
C13—C12—C11	122.0 (2)	C27—C26—H26A	109.9
C13—C12—H12	119.0	N7—C26—H26B	109.9
C11—C12—H12	119.0	C27—C26—H26B	109.9
C12—C13—C14	121.0 (2)	H26A—C26—H26B	108.3
C12—C13—Cl4	119.3 (2)	N9—C27—C28	121.6 (2)
C14—C13—Cl4	119.7 (2)	N9—C27—C26	115.59 (19)
C18—C14—C15	116.3 (3)	C28—C27—C26	122.7 (2)
C18—C14—C13	117.4 (2)	C29—C28—C27	118.6 (2)
C15—C14—C13	126.3 (3)	C29—C28—H28	120.7
C16—C15—C14	119.5 (3)	C27—C28—H28	120.7
C16—C15—H15	120.2	C30—C29—C28	119.5 (2)
C14—C15—H15	120.2	C30—C29—H29	120.3
C15—C16—C17	119.7 (3)	C28—C29—H29	120.3
C15—C16—H16	120.1	C29—C30—C31	118.9 (3)
C17—C16—H16	120.1	C29—C30—H30	120.6
N6—C17—C16	124.0 (3)	C31—C30—H30	120.6
N6—C17—H17	118.0	N9—C31—C30	122.1 (3)
C16—C17—H17	118.0	N9—C31—H31	118.9
N6—C18—C14	123.9 (2)	C30—C31—H31	118.9
N6—C18—C10	115.5 (2)		
			
O5—C10—C11—C12	−179.8 (2)	C26—N7—C20—C21	155.72 (18)
C18—C10—C11—C12	−0.7 (3)	C19—N7—C20—C21	−82.6 (2)
O5—C10—C11—C19	0.8 (3)	Zn3—N7—C20—C21	45.9 (2)
C18—C10—C11—C19	179.9 (2)	C25—N8—C21—C22	−0.2 (3)
C10—C11—C12—C13	1.3 (3)	Zn3—N8—C21—C22	179.88 (18)
C19—C11—C12—C13	−179.3 (2)	C25—N8—C21—C20	179.6 (2)
C11—C12—C13—C14	−0.3 (4)	Zn3—N8—C21—C20	−0.3 (3)
C11—C12—C13—Cl4	−178.28 (18)	N7—C20—C21—N8	−33.3 (3)
C12—C13—C14—C18	−1.4 (4)	N7—C20—C21—C22	146.4 (2)
Cl4—C13—C14—C18	176.63 (18)	N8—C21—C22—C23	1.6 (4)
C12—C13—C14—C15	179.1 (2)	C20—C21—C22—C23	−178.2 (2)
Cl4—C13—C14—C15	−2.9 (4)	C21—C22—C23—C24	−1.5 (4)
C18—C14—C15—C16	−1.0 (4)	C22—C23—C24—C25	0.2 (4)
C13—C14—C15—C16	178.5 (3)	C21—N8—C25—C24	−1.3 (4)
C14—C15—C16—C17	0.0 (5)	Zn3—N8—C25—C24	178.66 (19)
C18—N6—C17—C16	−0.5 (4)	C23—C24—C25—N8	1.3 (4)
C15—C16—C17—N6	0.8 (5)	C20—N7—C26—C27	−158.30 (18)
C17—N6—C18—C14	−0.7 (4)	C19—N7—C26—C27	77.8 (2)
C17—N6—C18—C10	179.2 (2)	Zn3—N7—C26—C27	−48.52 (19)
C15—C14—C18—N6	1.4 (4)	C31—N9—C27—C28	−0.5 (3)
C13—C14—C18—N6	−178.1 (2)	Zn3—N9—C27—C28	−176.89 (17)
C15—C14—C18—C10	−178.5 (2)	C31—N9—C27—C26	176.6 (2)
C13—C14—C18—C10	2.0 (3)	Zn3—N9—C27—C26	0.2 (2)
O5—C10—C18—N6	−1.7 (3)	N7—C26—C27—N9	35.0 (3)
C11—C10—C18—N6	179.1 (2)	N7—C26—C27—C28	−147.9 (2)
O5—C10—C18—C14	178.2 (2)	N9—C27—C28—C29	1.5 (4)
C11—C10—C18—C14	−1.0 (3)	C26—C27—C28—C29	−175.4 (2)
C20—N7—C19—C11	70.4 (3)	C27—C28—C29—C30	−1.0 (4)
C26—N7—C19—C11	−165.5 (2)	C28—C29—C30—C31	−0.3 (4)
Zn3—N7—C19—C11	−49.1 (2)	C27—N9—C31—C30	−0.9 (4)
C10—C11—C19—N7	−96.5 (3)	Zn3—N9—C31—C30	175.09 (18)
C12—C11—C19—N7	84.1 (3)	C29—C30—C31—N9	1.3 (4)

### Hydrogen-bond geometry (Å, º)

**Table:**

*D*—H···*A*	*D*—H	H···*A*	*D*···*A*	*D*—H···*A*
O5—H5···N6	0.79 (4)	2.14 (4)	2.653 (3)	124 (3)
C16—H16···Br2^i^	0.95	2.87	3.808 (3)	170
C22—H22···Br2^ii^	0.95	2.88	3.581 (3)	131
C29—H29···Br1^iii^	0.95	2.90	3.798 (3)	158

Symmetry codes: (i) −*x*+1, −*y*, −*z*; (ii) *x*+1, *y*−1, *z*; (iii) −*x*+1, −*y*+1, −*z*+1.
